# Effect of carnitine on muscular glutamate uptake and intramuscular glutathione in malignant diseases

**DOI:** 10.1054/bjoc.1999.0933

**Published:** 2000-01-18

**Authors:** R Breitkreutz, A Babylon, V Hack, K Schuster, M Tokus, H Böhles, E Hagmüller, L Edler, E Holm, W Dröge

**Affiliations:** Division of Immunochemistry, Deutsches Krebsforschungszentrum, In Neuenheimer Feld 280, Heidelberg, D-69120, Germany; Chirurgische Klinik, Manheim, D-68167, Germany; Abt. für Pathophysiologie, 1. Medizinische Klinik, Mannheim, D-68167, Germany; Universitäts-Kinderklinik, Universitätsklinik Frankfurt am Main, Germany; Biostatistics Unit, Deutsches Krebsforschungszentrum, Heidelberg, D-69120, Germany

**Keywords:** carnitine, glutathione, glutamate transport, amino acid exchange rates

## Abstract

Abnormally low intramuscular glutamate and glutathione (GSH) levels and/or a decreased muscular uptake of glutamate by the skeletal muscle tissue have previously been found in malignant diseases and simian immunodeficiency virus (SIV) infection and may contribute to the development of cachexia. We tested the hypothesis that an impaired mitochondrial energy metabolism may compromise the Na^+^-dependent glutamate transport. A randomized double-blind clinical trial was designed to study the effects of L -carnitine, i.e. an agent known to enhance mitochondrial integrity and function, on the glutamate transport and plasma glutamate level of cancer patients. The effect of carnitine on the intramuscular glutamate and GSH levels was examined in complementary experiments with tumour-bearing mice. In the mice, L -carnitine treatment ameliorated indeed the tumour-induced decrease in muscular glutamate and GSH levels and the increase in plasma glutamate levels. The carnitine-treated group in the randomized clinical study showed also a significant decrease in the plasma glutamate levels but only a moderate and statistically not significant increase in the relative glutamate uptake in the lower extremities. Further studies may be warranted to determine the effect of L -carnitine on the intramuscular GSH levels in cancer patients. © 2000 Cancer Research Campaign


*British Journal of Cancer* (2000) **82**(2), 399–403
© 2000 Cancer Research Campaign
Article no. bjoc.1999.0933

### Effect of carnitine on muscular glutamate uptake and intramuscular glutathione in malignant diseases

**R Breitkreutz[1], A Babylon[1], V Hack[1], K Schuster[2], M Tokus[3], H Böhles[4], E Hagmüller[2], L Edler[5], E Holm[3] and W Dröge[1]**

[1]Deutsches Krebsforschungszentrum, Division of Immunochemistry, In Neuenheimer Feld 280, D-69120 Heidelberg, Germany; [2]Chirurgische Klinik, D-68167 Mannheim, Germany; [3]Abt. für Pathophysiologie, 1. Medizinische Klinik, D-68167 Mannheim, Germany; [4]Universitäts-Kinderklinik, Universitätsklinik Frankfurt am Main, Germany; [5]Biostatistics Unit, Deutsches Krebsforschungszentrum, D-69120 Heidelberg, Germany

**Summary** Abnormally low intramuscular glutamate and glutathione (GSH) levels and/or a decreased muscular uptake of glutamate by the skeletal muscle tissue have previously been found in malignant diseases and simian immunodeficiency virus (SIV) infection and may contribute to the development of cachexia. We tested the hypothesis that an impaired mitochondrial energy metabolism may compromise the $Na^+$-dependent glutamate transport. A randomized double-blind clinical trial was designed to study the effects of L-carnitine, i.e. an agent known to enhance mitochondrial integrity and function, on the glutamate transport and plasma glutamate level of cancer patients. The effect of carnitine on the intramuscular glutamate and GSH levels was examined in complementary experiments with tumour-bearing mice. In the mice, L-carnitine treatment ameliorated indeed the tumour-induced decrease in muscular glutamate and GSH levels and the increase in plasma glutamate levels. The carnitine-treated group in the randomized clinical study showed also a significant decrease in the plasma glutamate levels but only a moderate and statistically not significant increase in the relative glutamate uptake in the lower extremities. Further studies may be warranted to determine the effect of L-carnitine on the intramuscular GSH levels in cancer patients. © 2000 Cancer Research Campaign

**Keywords:** carnitine; glutathione; glutamate transport; amino acid exchange rates

Several studies on aetiologically unrelated catabolic conditions suggest that the massive loss of skeletal muscle mass (cachexia) is associated with a decrease in the intracellular glutathione (GSH) level in the muscle tissue. Decreased GSH levels and increased glutathione disulphide/glutathione (GSSG/GSH) ratios have been observed in skeletal muscle of weight-losing tumour-bearing mice (Hack et al, 1996*a*) and rhesus monkeys infected with the simian immunodeficiency virus (SIV) (Groß et al, 1996), i.e. the best experimental animal model of AIDS. Unless cysteine or glycine or the corresponding biosynthetic enzymes become limiting, the GSH level is determined by the glutamate level, because GSH competes with glutamate for the glutamate-binding site of the γ-glutamyl-cysteine synthetase in the first and rate-limiting step of GSH biosynthesis (Richman and Meister, 1975). The skeletal muscle tissue of tumour-bearing mice and SIV-infected monkeys showed indeed a decreased intracellular glutamate level (Groß et al, 1996; Hack et al, 1996*a*).

Several lines of evidence suggest that the decrease in the intramuscular glutamate level results from a decreased glutamate transport activity across the plasma membrane. First, studies of amino acid exchange rates across the lower extremities revealed that the relative glutamate uptake of cancer patients is significantly decreased (Hack et al, 1996*b*). Secondly, the decrease in the muscular glutamate level of tumour-bearing mice and SIV-infected rhesus macaques was found to be associated with a strong increase in the plasma glutamate level (Groß et al, 1996, Hack et al, 1996*a*).

*Received 10 December 1998*
*Revised 30 June 1999*
*Accepted 5 July 1999*

*Correspondence to:* W Dröge, E-mail: W.Droege@dkfz-heidelberg.de

And finally, increased post-absorptive venous plasma glutamate levels have been found in all catabolic conditions tested so far including malignancies (Brennan, 1977; Dröge et al, 1988*a*, 1988*b*; Pisters and Pearlstone, 1993), HIV/SIV infection (Eck et al, 1989, 1991), sepsis (Siegel et al, 1979), non-insulin-dependent diabetes mellitus (Hack et al, 1996*a*), amyotrophic lateral sclerosis (Plaitakis and Caroscio, 1987) and old age (Hack et al, 1996*b*). Even in healthy human subjects, it was observed that episodes with elevated plasma glutamate levels were significantly correlated with a decrease in body cell mass (Kinscherf et al, 1996). A study of lung cancer patients revealed that plasma glutamate levels were significantly correlated with mortality (Eck et al, 1989). The impairment of the glutamate uptake appears to be a relatively early event in the development of cachectic processes, because increased plasma glutamate levels are found already in the very early stages of HIV and SIV infection (Eck et al, 1991; Groß et al, 1996; Hack et al, 1997), and a decrease in glutamate uptake was found already in well nourished cancer patients (Hack et al, 1996*b*).

Because glutamate is transported into muscle cells mainly by a $Na^+$-dependent membrane transport system (Horn 1989; Low et al, 1994; McGivan and Pastor-Anglada, 1994), and in view of the link between $Na^+$ gradient and intracellular pH via the $Na^+/H^+$ antiport, we consider the working hypothesis that decreased glutamate transport activity may be an indirect consequence of abnormally high glycolytic activity. An increased glycolytic activity has been reported to occur quite regularly and relatively early in malignancies, burn injuries and sepsis (Wilmore and Aulick, 1978; Roth et al, 1982; Striebel et al, 1986; Shaw and Wolfe, 1987; Tayek, 1992). A high glycolytic activity and lactate production is an indication that the capacity of the mitochondrial energy metabolism is too weak to meet the cellular demand for ATP.

**Table 1** Baseline characteristics[a] of the patients in the randomized double-blind trial

| | **L-carnitine (*n* = 14)** | **Placebo (*n* = 14)** |
|---|---|---|
| Male/female | 10/4 | 8/6 |
| Age (years)[b] | 64.0±9.9 | 64.3±9.3 |
| Percentage of optimimal body weight[b] | 116.7±16.6 | 105.4±10.8 |
| Body cell mass (BCM) index[b,c] (male/female) (kg m$^{-2}$) | 9.2±2.3 (10.2/6.8) | 8.3±1.7 (9.3/7.1) |
| Tumour type | | |
| Gastrointestinal (*n*) | 10 | 10 |
| Renal cancer (*n*) | 1 | 2 |
| Others (*n*) | 3 | 2 |
| Free carnitine | | |
| Arterial µM | 30.3±2.6 | 32.1±2.7 (30.1±1.8)[d] |
| Venous µM | 34.2±2.2 | 33.2±2.7 (31.1±1.8)[d] |
| Acyl-carnitine[c] | | |
| Arterial µM | 16.2±3.3 | 9.7±1.7 (10.1±0.9)[d] |
| Venous µM | 14.7±2.2 | 11.4±1.4 (10.2 ± 1.3)[d] |

[a] Electrolytes and routine liver and kidney parameters were in the normal range. [b]Mean ± standard error of the mean (s.e.m.). [c]BCM height$^{-2}$; mean of healthy controls (m/f): 10.8 ± 1.8 (11.9/8.8) kg m$^{-2}$. [d]Mean values ± s.e.m. of a healthy age-matched control population (*n* = 14) is given in brackets.

L-carnitine plays an important role in the mitochondrial energy metabolism because it serves as a carrier for fatty acids across the inner mitochondrial membrane (Bremer, 1983). In several studies, carnitine or acetyl-carnitine were found to enhance mitochondrial integrity and function (Nikula, 1985; McFalls et al, 1986; Hagen et al, 1998). This effect is attributed mainly to the transfer of inner mitochondrial acyl-moieties (Rebouche and Paulson, 1986). Two investigations on cancer patients revealed that the patients under study had an abnormally high renal clearance of carnitine (Dodson et al, 1989) and a significantly decreased carnitine level in the skeletal muscle tissue respectively (Rössle et al, 1989). Moreover, treatment of tumour-bearing rats was shown to ameliorate the tumour-induced increase in plasma triglycerides (Winter et al, 1995). We, therefore, studied the hypothesis that administration of L-carnitine to cancer patients and tumour-bearing mice may ameliorate the decrease in glutamate uptake, intracellular glutamate and GSH levels in the skeletal muscle tissue respectively.

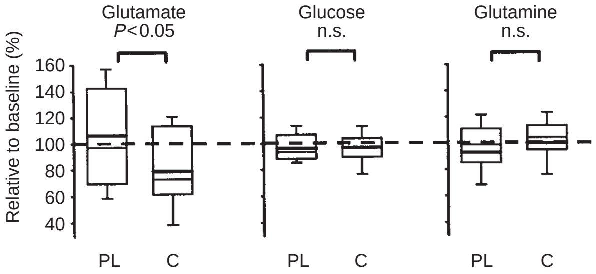

**Figure 1** Effect of L-carnitine or placebo on the relative change in plasma glutamate, glucose and glutamine. The data show the median (strong line) and the mean (thin line) of the plasma concentrations at terminal examination expressed as % of the corresponding individual baseline values. The box plot describes, in addition, the first (25%) and third (75%) quartile of the distribution. The *P*-value is given for the difference between the L-carnitine group (C) and the placebo group (PL). The dashed line indicates no change (100%)

### PATIENTS, MATERIALS AND METHODS

### Randomized double-blind clinical trial

Well nourished patients with carcinoma of the stomach, the colon, the liver, the pancreas, the kidney or the lung were eligible. Patients younger than 20 or older than 75 years, patients who were undergoing surgery within 3 months before recruitment and patients who had already received chemotherapy or radiation therapy were not eligible. Also excluded were patients with diabetes mellitus or other endocrinological diseases, multiple sclerosis, serum creatinine > 2 mg dl$^{-1}$, cardio-respiratory insufficiency, alcohol or drug abuse, anaemia, or pregnant women. Clinical laboratory data including the electrolytes, glucose, total bilirubin, creatinine, ALT, cholesterol and triglycerides were in the normal range. Twenty-eight patients were recruited and randomly assigned to the L-carnitine (*n* = 14) and placebo (*n* = 14) groups. Randomization was performed by the biostatistics unit of the German Cancer Research Center (LE). The study was approved by the ethics committee of the Medical Faculty of the University of Heidelberg/Mannheim and was conducted according to the GCP-ICH/GLP guidelines and to the principles of the declaration of Helsinki. All patients gave their informed consent.

The patients received a daily oral dose of 2 g L-carnitine or placebo dissolved in fruit juice for 5 consecutive days and were examined twice, i.e. on the first day of treatment and 5 days after the start of treatment. The placebo contained propyl-4-hydroxy benzoate, sorbitol and lemon flavouring. The estimated dietary carnitine intake was approximately 50 mg day$^{-1}$. Blood samples were taken in the post-absorptive state from the femoral artery and vein. Additional blood samples were taken from the cubital vein for routine clinical parameters. Carnitine and carnitine-esters were determined as described by McGarry and Foster (1976). Plasma amino acid concentrations, plasma glucose, lactate and ketone bodies were determined as described by Hack et al (1996*b*). Prospectively defined primary outcome measure was the glutamate exchange rate. Because the glutamate uptake was previously shown to depend strongly on the arterial glutamate concentration, we expressed the data as 'relative glutamate uptake' (i.e. the ratio of glutamate uptake/arterial glutamate level) as defined by Hack et al (1996*b*). Secondary outcome measures were the venous plasma glutamate level and the exchange rates of glutamine and glucose. Plasma amino acid and whole blood glucose exchange rates were determined as described (Hack et al, 1996*b*).

The relative changes of the outcome parameters of the two different treatment groups were compared by the Wilcoxon rank-sum-test. *P*-values <0.05 were regarded as statistically significant.

*© 2000 Cancer Research Campaign*

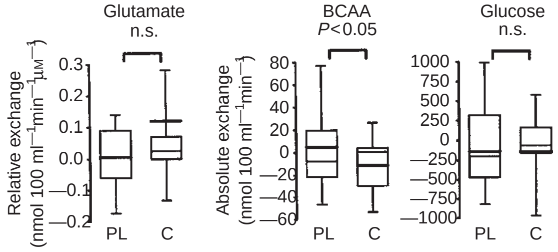

**Figure 2** Effect of L-carnitine or placebo on the exchange rates of plasma glutamate, glucose and branched-chain amino acids. The data show the mean of the individual differences between the measurements at terminal and baseline examination. For other details see legend to Figure 1. Because the glutamate uptake was previously shown to depend strongly on the arterial glutamate concentration, we indicated in Figure 2 the changes in the 'relative glutamate uptake' as defined by the ratio of glutamate uptake/arterial glutamate level (see Hack et al, 1996*b*)

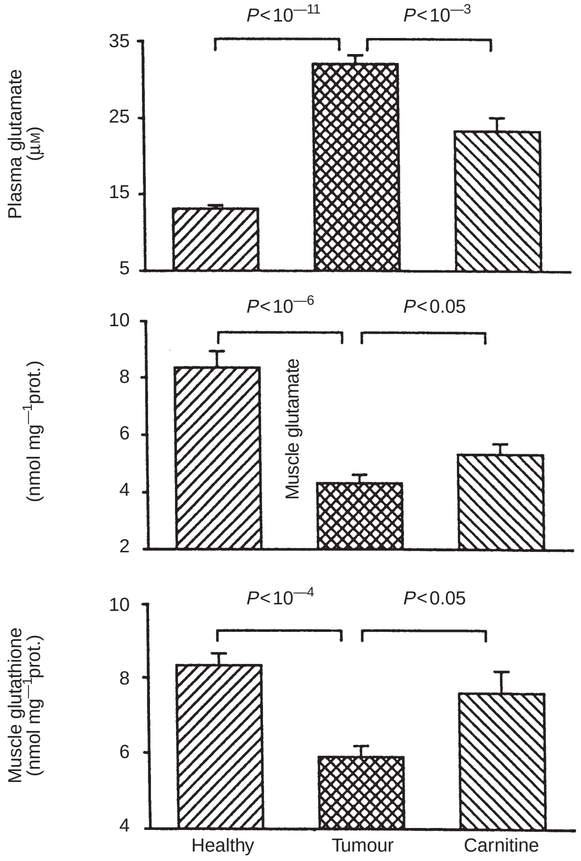

**Figure 3** Effect of L-carnitine on the intramuscular GSH and glutamate levels and plasma glutamate concentrations of tumour-bearing mice. The bars show the results (arithmetic means ± s.e.m.) from normal mice, tumour-bearing mice and tumour-bearing mice treated with a dose of 1 mg L-carnitine i.p. per day. The data show the means ± s.e.m. from 24 separate muscle preparations each (i.e. two separate experiments with six animals per group and two separate muscle preparations per mouse)

Arithmetic means and standard errors of the mean (s.e.m.) were used as descriptive statistics.

### L-carnitine treatment of tumour-bearing mice

Female C57BL/6 mice were obtained from the Central Animal Laboratories of the German Cancer Research Center (DKFZ), Heidelberg. The mice were fed ad libitum with a standard diet (Altromin) and were usually 10–16 weeks old at the start of the experiment. Altromin containes 3% 'fishprotein' (Dr Madry, Altromin, personal communication). 'Fishprotein', in turn, was reported to contain 1.5 μmol of L-carnitine $g^{-1}$ wet weight (*http://galaxy.com/galaxy/Community/Health/Diet/carnitine/food.htm*). The MCA-105 fibrosarcoma was originally induced in a C57BL/6 mouse by intramuscular injection of methylcholanthrene (Fox et al, 1990). In the present study, the mice received $3 \times 10^6$ tumour cells in 0.1 ml HBSS subcutaneously (day 0) and daily intraperitoneal injections of 0.4 ml HBSS containing either 0.0, 0.04, 0.2, 1.0 or 5.0 mg L-carnitine on days 11–15 and 18–22. As a rule, the tumour was barely palpable on day 11 and had a diameter of about 1 cm on day 22. The mice were not cachectic. L-carnitine had no significant effect on the tumour size.

The mice were bled from the tail vein. Blood was collected in heparinized tubes, and the plasma amino acid concentrations were determined with an amino acid analyser (Biotronic LC 3000) as described (Hack et al, 1996*a*).

*M. gastrocnemius* and *M. vastus lateralis* were prepared separately and further dissected into a red section (muscle type 2A) and a white section (type 2B). The white sections were stored at –80°C, and pulverized in liquid nitrogen. Samples of liver or muscle powder were transferred into 0.4 ml 2.5% sulphosalicylic acid, subjected to sonification and finally kept on ice for at least 20 min. The mixture was centrifuged for 10 min at 12 000 ***g***, and the supernatants were used for the determination of amino acids (Biotronic LC 3000 amino acid analyser), total glutathione according to Tietze (1969), and glutathione disulphide according to Griffith (1980). The corresponding pellet was used to determine the protein content by the procedure of Peterson (1977).

### RESULTS

### A placebo-controlled double-blind trial on the effects of L-carnitine

The baseline characteristics of the two treatment groups were not significantly different (Table 1), and there was no significant change in the plasma levels of carnitine or carnitine esters during the treatment (not shown). The glutamate exchange rates of the L-carnitine and placebo groups at baseline examination were expectedly relatively low (i.e. 48.3 ± 8.7 and 39.2 ± 7.7 $nmol^{-1}$ $min^{-1}$ 100 $ml^{-1}$) (compare Hack et al, 1996*b*). The carnitine-treated group showed, on average, a significant decrease in the venous plasma glutamate level but only a moderate and statistically not significant increase in the relative glutamate uptake in the lower extremities (Figure 1 and 2). In addition, L-carnitine treatment increased the release of BCAA in comparison with the placebo group but had no detectable effect on the plasma glutamine or glucose levels (Figure 1) and on the muscular glucose uptake (Figure 2).

The treatment was well tolerated. No serious side-effects or subjective changes have been reported.

### Effects of L-carnitine on the glutamate and GSH levels in tumour-bearing mice

Because the clinical study did not allow us to determine the resulting changes in the intracellular GSH and glutamate levels of the skeletal muscle tissue, we also studied the effect of carnitine on tumour-bearing mice.

© 2000 Cancer Research Campaign

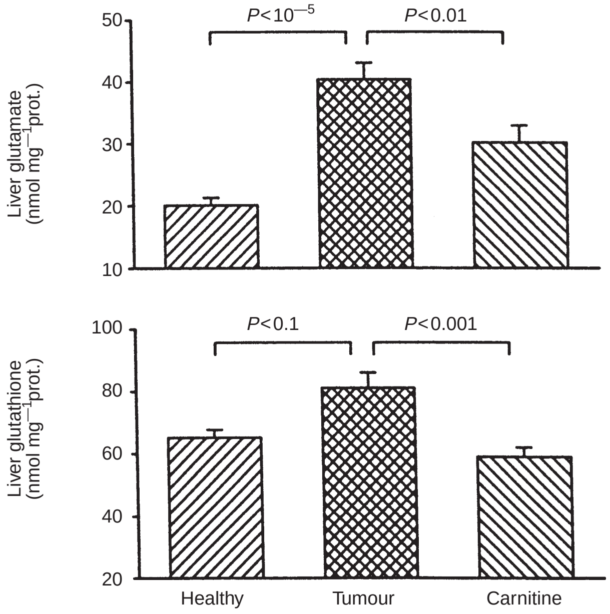

**Figure 4** Effect of L-carnitine on the GSH and glutamate levels in the liver of tumour-bearing mice. For details see legend to Figure 3

C57/BL6 mice bearing the MCA 105 fibrosarcoma (Hack et al, 1996*a*) received daily intraperitoneal injections of graded doses of L-carnitine (i.e. 0.0, 0.04, 0.2, 1, or 5 mg per day) on 7 consecutive days starting 2 weeks after tumour inoculation. Mice were sacrificed 1 day after the last injection of L-carnitine. The results confirmed the earlier findings that intramuscular GSH and glutamate concentrations in the tumour-bearing mice are strongly decreased and plasma glutamate concentrations strongly increased in comparison with non-tumour-bearing controls. These changes were at least partly reversed by daily injections of 1 mg L-carnitine (Figure 3). Lower doses yielded no significant changes, whereas 5 mg day$^{-1}$ (approximately 250 mg kg$^{-1}$ day$^{-1}$) reversed the increase in intramuscular glutamate and glutathione that was seen with 1 mg day$^{-1}$ (not shown). The changes in the blood plasma were also reflected in corresponding changes in the liver (Figure 4). These changes were essentially the mirror image of the changes in the skeletal muscle tissue. However, L-carnitine induced no consistent changes in the levels of glutamine or BCAA in the plasma or skeletal muscle tissue of the tumour-bearing mice (data not shown).

### DISCUSSION

The results from the tumour-bearing mice confirm the hypothesis that L-carnitine may be an effective treatment to reconstitute the abnormally low intramuscular GSH levels in malignancies. The concomitant increase in the intramuscular glutamate and the decrease in the plasma glutamate level in carnitine-treated mice support the interpretation that the increase in muscular GSH may be mediated by an increase in the net glutamate transport into the skeletal muscle tissue. In agreement with these findings, carnitine treatment caused also a significant decrease in plasma glutamate levels in our randomized study on cancer patients, although the relative net glutamate uptake in the lower extremities of the cancer patients was only marginally increased by this treatment. To explain this apparent discrepancy we propose tentatively that ingestion of L-carnitine may enhance the glutamate transport only transiently. This effect may not be detectable any more in the post-absorptive period, when the decrease in the plasma glutamate level, i.e. the putative consequence of the temporary increase in glutamate uptake, is still demonstrable. This interpretation remains to be tested. The effect of L-carnitine on the release of BCAA (Figure 2), finally, is still poorly understood and requires further investigation.

Elevated hepatic GSH levels in tumour-bearing mice (Figure 4) may appear as a compensatory antioxidant response and may seem advantageous at face value. However, earlier investigations on this tumour model have shown that the increase in hepatic GSH is associated with an increased release of GSH into the bile and may therefore lead ultimately to a loss of cysteine (Hack et al, 1996*a*). It is tempting to assume that the increased hepatic glutamate and GSH levels may result indirectly from the decreased glutamate uptake by the skeletal muscle tissue and the corresponding increase in plasma glutamate levels. However, it cannot be formally excluded that biochemical changes in the liver itself may contribute to the systemic increase in glutamate levels.

Taken together, the effects of L-carnitine on the intramuscular GSH levels is potentially useful in clinical therapy. This therapeutic potential and especially the effects of longer treatment periods may deserve further investigation. However, high doses of L-carnitine, i.e. 250 mg kg$^{-1}$ day$^{-1}$, may be disadvantageous, because this dose was found to cause a substantial decrease in intramuscular glutamate and GSH levels in the skeletal muscle tissue of tumour-bearing mice.

### ACKNOWLEDGEMENTS

The assistance of Mrs I Fryson in the preparation of this manuscript is gratefully acknowledged. We also thank Mrs U Winter, N Erbe and Mr H Lips for the dedicated technical assistance, and Dr R Fischer, Medice, Iserlohn, for the study medication.

### REFERENCES

Bremer J (1983) Carnitine – metabolism and functions. *Physiol Rev* **63**: 1420–1480

Brennan MF (1977) Uncomplicated starvation versus cancer cachexia. *Cancer Res* **37**: 2359–2364

Dodson WL, Sachan DS, Krauss S and Hanna W (1989) Alterations of serum and urinary carnitine profiles in cancer patients: hypothesis of possible significance. *J Am Coll Nutr* **8**: 133–142

Dröge W, Eck H-P, Betzler M, Schlag P, Drings P and Ebert W (1988*a*) Plasma glutamate concentration and lymphocyte activity. *J Cancer Clin Oncol* **114**: 124–128

Dröge W, Eck H-P, Näher H, Pekar U and Daniel V (1988*b*) Abnormal amino acid concentrations in the blood of patients with acquired immune deficiency syndrome (AIDS) may contribute to the immunological defect. *Biol Chem Hoppe-Seyler* **369**: 143–148

Eck H-P, Drings P and Dröge W (1989) Plasma glutamate levels, lymphocyte reactivity and death rate in patients with bronchial carcinoma. *J Cancer Clin Oncol* **115**: 571–574

Eck H-P, Stahl-Hennig C, Hunsmann G and Dröge W (1991) Metabolic disorder as an early consequence of simian immunodeficiency virus infection in rhesus macaques. *Lancet* **338**: 346–347

Fox BA, Spiess PJ, Kasid A, Puri R, Mulé JJ, Weber JS and Rosenberg SA (1990) In vitro and in vivo antitumor properties of a T cell clone generated from murine tumor-infiltrating lymphocytes. *J Biol Response Mod* **9**: 499–511

Griffith OW (1980) Determination of glutathione and glutathione disulfide using glutathione reductase and 2-vinylpyridine. *Anal Biochem* **106**: 207–212

Groß A, Hack V, Stahl-Henning C and Dröge W (1996) Elevated hepatic γ-glutamylcysteine synthetase activity and abnormal sulfate levels in liver and

© 2000 Cancer Research Campaign muscle tissue may explain abnormal cysteine and glutathione levels in SIV-infected rhesus macaques. *AIDS Res Hum Retrovirus* **12**: 1639–1641
Hack V, Gross A, Kinscherf R, Bockstette M, Fiers W, Berke G and Dröge W (1996*a*) Abnormal glutathione and sulfate levels after interleukin 6 treatment and in tumor-induced cachexia. *FASEB J* **10**: 1219–1226
Hack V, Stütz O, Kinscherf R, Schykowski M, Kellerer M, Holm E and Dröge W (1996*b*) Elevated venous glutamate levels in (pre)catabolic conditions result at least partly from a decreased glutamate transport activity. *J Mol Med* **74**: 337–343
Hack V, Schmidt D, Breitkreutz R, Stahl-Henning C, Kinscherf R, Taut F, Holm E and Dröge W (1997) Cystine levels, cystine flux, and protein catabolism in cancer cachexia, HIV/SIV infection, and senescence. *FASEB J* **11**: 84–92
Hagen TM, Ingersoll RT, Wehr CM, Lykkesfeldt J, Vinarsky V, Bartholomew JC, Song M-H and Ames BN (1998) Acetyl-L-carnitine fed to old rats partially restores mitochondrial function and ambulatory activity. *Proc Natl Acad Sci USA* **95**: 9562–9566
Horn LW (1989) L-Glutamate transport in internally dialysed barnacle muscle fibres. *Am J Physiol* **257**: C442–C450
Kinscherf R, Hack V, Fischbach T, Friedmann B, Weiss C, Edler L, Bärtsch P and Dröge W (1996) Low plasma glutamine in combination with high glutamate levels indicate risk for loss of body cell mass in healthy individuals: the effect of N-acetyl-cysteine. *J Mol Med* **74**: 393–400
Low SY, Rennie MJ and Taylor PM (1994) Sodium-dependent glutamate transport in cultured rat myotubes increases after glutamate deprivation. *FASEB J* **8**: 127–131
McFalls EO, Paulson DJ, Gilbert EF and Shug AL (1986) Carnitine protection against adriamycin-induced cardiomyopathy in rats. *Life Sci* **38**: 497–505
McGarry JD and Foster DW (1976) An improved and simplified radioisotopic assay for the determination of free and esterified carnitine. *J Lipid Res* **17**: 277–281
McGivan JD and Pastor-Anglada M (1994) Regulatory and molecular aspects of mammalian amino acid transport. *Biochem J* **299**: 321–334
Nikula P, Ruohola H, Alhonen-Hongisto L and Janne J (1985) Carnitine prevents the early mitochondrial damage induced by methylglyoxal bis(guanylhydrazone) in L1210 leukemia cells. *Biochem J* **228**: 513–516
Peterson GL (1977) A simplification of the protein assay method of Lowry et al which is more generally applicable. *Anal Biochem* **83**: 346–356
Pisters PWT and Pearlstone DB (1993) Protein and amino acid metabolism in cancer cachexia: lnvestigative techniques and therapeutic interventions. *Crit Rev Clin Lab Sci* **30**: 223–272
Plaitakis A and Caroscio JT (1987) Abnormal glutamate metabolism in amyotrophic lateral sclerosis. *Ann Neurol* **22**: 575–579
Rebouche CJ and Paulson DS (1986) Carnitine metabolism and function in humans. *Ann Rev Nutr* **6**: 41–66
Richman PG and Meister A (1975) Regulation of γ-glutamyl-cysteine synthetase by nonallosteric feedback inhibition by glutathione. *J Biol Chem* **250**: 1422–1426
Rössle C, Pichard C, Roulet M, Bergström J and Fürst P (1989) Muscle carnitine pools in cancer patients. *Clin Nutr* **8**: 341–346
Roth E, Funovics J, Mühlbacher F, Schemper M, Mauritz W, Sporn P and Fritsch A (1982) Metabolic disorders in severe abdominal sepsis: glutamine deficiency in skeletal muscle. *Clin Nutr* **1**: 25–41
Shaw JH and Wolfe RR (1987) Glucose and urea kinetics in patients with early and advanced gastrointestinal cancer: the response to glucose infusion and TPN. *Surgery* **101**: 181–186
Striebel J-P, Saeger H-D, Ritz R, Leweling H and Holm E (1986) Aminosäureaufnahme und-abgabe kolorektaler Karzinome des Menschen. *Infusionstherupie* **13**: 92–104
Tayek JA (1992) A review of cancer cachexia and abnormal glucose metabolism in humans with cancer. *J Am Col Nutr* **11**: 445–456
Tietze F (1969) Enzymic method for quantitative determination of nanogram amounts of total and oxidized glutathione: applications to mammalian blood and other tissues. *Anal Biochem* **27**: 502–522
Wilmore DW and Aulick LH (1978) Metabolic changes in burned patients. *Surg Clin N Am* **58**: 1173–1187
Winter BK, Fiskum G and Gallo LL (1995) Effects of L-carnitine on serum triglyceride and cytokine levels in rat models of cachexia and septic shock. *Br J Cancer* **72**: 1173–1179

*© 2000 Cancer Research Campaign*
